# Case Report: Basal ganglia brain abscess caused by *Nocardia farcinica*

**DOI:** 10.3389/fmed.2026.1798434

**Published:** 2026-05-14

**Authors:** Feiwen Liu, Ke Yang, Mengna Wu, Pan Li, Lun Luo

**Affiliations:** West China School of Medicine, Sichuan University, Sichuan University Affiliated Chengdu Second People’s Hospital, Chengdu Second People’s Hospital, Chengdu, Sichuan, China

**Keywords:** brain abscess, hemiplegia, immunocompromised, metagenomics next generation sequencing (mNGS), *Nocardia farcinica*

## Abstract

We report a rare case of *Nocardia farcinica* brain abscess in the basal ganglia, detailing its diagnosis, management, and rehabilitation. Diagnosing brain abscess based solely on clinical and imaging findings remains extremely challenging. Fortunately, metagenomic next-generation sequencing (mNGS) proved valuable in this case by rapidly identifying the pathogen, thereby facilitating targeted antibiotic therapy. This case highlights the importance of differentiating brain abscess from ischemic stroke and intracranial tumors. After completing a full course of anti-infective therapy and comprehensive rehabilitation, the patient achieved significant recovery in activities of daily living (ADL).

## Background

Brain abscesses have an incidence of 0.8 per 100,000 population and a mortality rate of approximately 10% (1, 2). *Nocardia farcinica* brain abscesses are particularly rare, accounting for only about 2% of all brain abscesses, with 62% occurring in immunocompromised individuals (3). Critically, their mortality rate is three times higher than that of other bacterial brain abscesses (30% vs. 10%) (4), making early diagnosis and treatment essential for improving outcomes.

Diagnosis remains challenging due to the variability of clinical presentation; only 14% of patients present with the classic triad of headache, fever, and focal neurological deficits (5). Even with advanced imaging, it can be difficult to reliably distinguish abscesses from ischemic strokes, brain tumors, or other intracranial lesions (6). Furthermore, reliance on cerebrospinal fluid or blood cultures can delay diagnosis, and false-negative results are not uncommon (7). These factors contribute to frequent misdiagnosis and delays in initiating appropriate treatment (8).

Here, we report a case of *Nocardia farcinica* brain abscess in the basal ganglia, accompanied by pulmonary nocardiosis and conjunctivitis. By detailing the diagnosis, management, and rehabilitation process, this case aims to enhance clinical recognition and inform the treatment of such rare and complex infections.

## Case presentation

**FIGURE 1:**
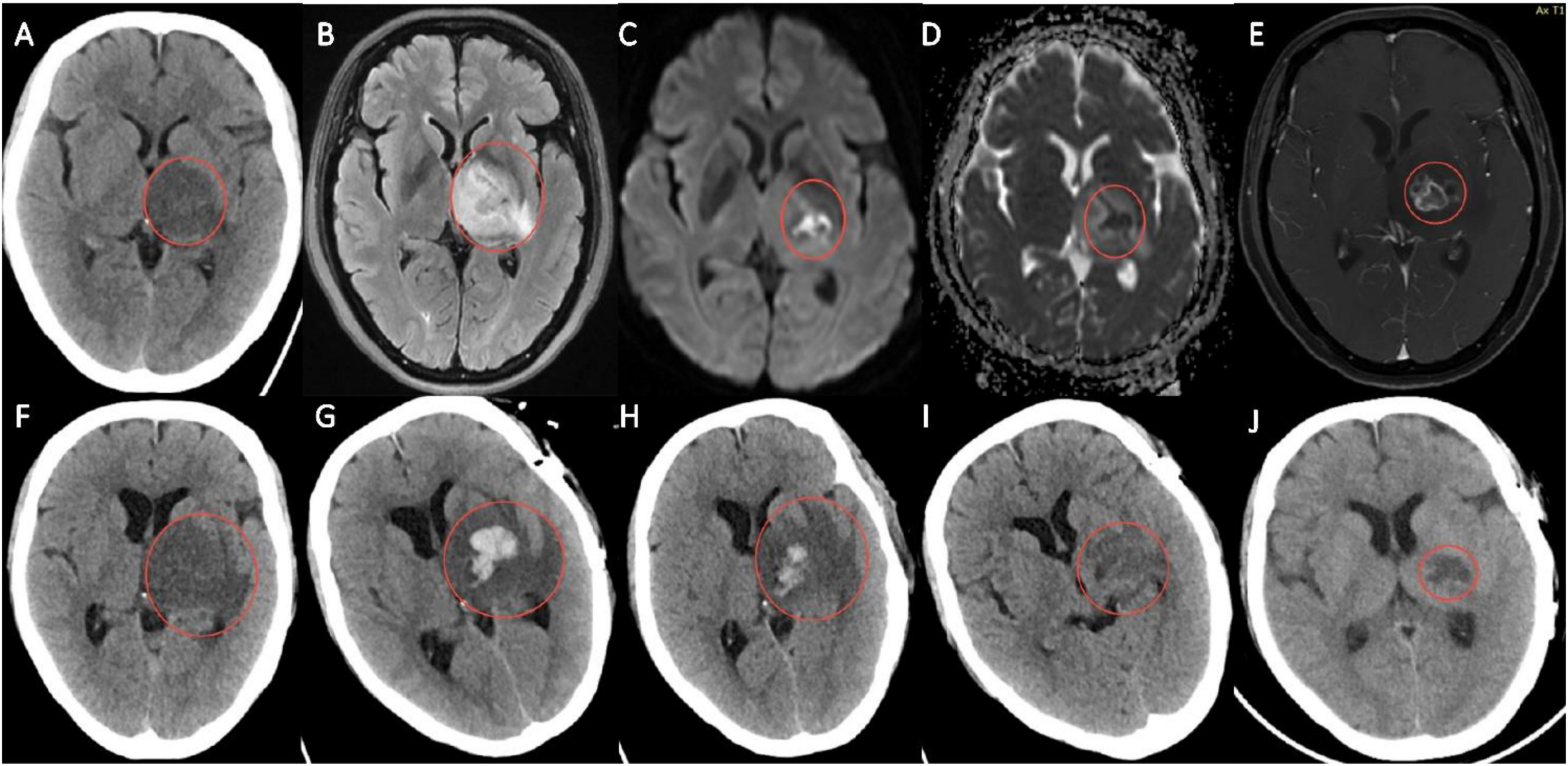
**(A)** Head CT at admission. **(B)** Head MRI scan. **(C,D)** Head Diffusion-weighted imaging (DWI) scan. **(E)** Contrast-enhanced MRI. **(F)** Head CT scan at the point of disease progression. **(G)** Post-operative head CT scan. **(H–J)** Follow-up head CT.

**FIGURE 2:**
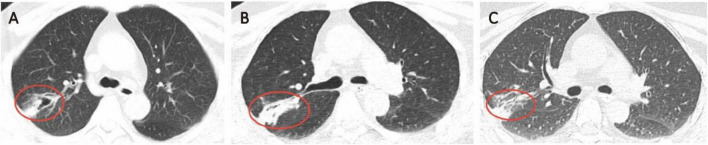
**(A)** Chest CT at admission. **(B,C)** Follow-up Chest CT.

A 55-years-old woman was admitted to the emergency department. Two days prior to admission, she developed acute right limb weakness that persisted without improvement. She worked in a furniture factory and had daily exposure to dust. Seven months before symptom onset, the patient had undergone a renal biopsy at another institution and was diagnosed with chronic kidney disease. Subsequently, she began daily immunosuppressive therapy with 32 mg of methylprednisolone. Physical examination revealed clear consciousness, grade 4 muscle strength in the right limb, normal muscle tone, tendon reflexes, sensation, coordination, and no pathological or meningeal signs. Head CT showed decreased density in the left paraventricular region, basal ganglia, thalamus, and cerebral peduncle. The midline structure was shifted to the right (Figure 1A). Chest CT revealed multiple lung infections, including consolidation with cavitation in the right lung (Figure 2A). Sputum culture showed a small amount of gram-negative bacilli and gram-positive cocci (in grape-like and chain-like forms). The patient had been diagnosed with nephrotic syndrome 4 months earlier and was taking oral methylprednisolone. An initial diagnosis of cerebral infarction was made, and antiplatelet therapy (aspirin combined with clopidogrel), intracranial pressure reduction (20% mannitol injection), and neurotrophic therapy were initiated.

On the 3rd day after admission, head MRI demonstrated infarct-like lesions with possible hemorrhage or neoplasm involving the left paraventricular area, basal ganglia, thalamus, cerebral peduncle, brainstem, and middle cerebellar peduncle (Figure 1B). Local regions of restricted diffusion were identified in the posterior limb of the internal capsule and the thalamic area. These regions were characterized by high signal intensity in diffusion-weighted imaging (DWI) (Figure 1C), and low signal intensity in the apparent diffusion coefficient (ADC) map (Figure 1D). Magnetic resonance angiography (MRA) indicated early bifurcation of the left middle cerebral artery, while MR venography (MRV) showed thinning and shallow segments of the left transverse sinus and inferior sagittal sinus. Susceptibility-weighted imaging (SWI) suggested minor hemorrhage in the right frontal lobe. Therefore, clopidogrel was discontinued. On the 5th day after admission, contrast-enhanced MRI and MR spectroscopy showed irregular ring enhancement in the left basal ganglia and thalamus, raising suspicion of high-grade glioma (Figure 1E). Cerebral infarction was ruled out, so aspirin was also discontinued. Surgical intervention was planned.

However, the patient’s condition deteriorated rapidly, with right limb strength declining from grade 4 to grade 1. Repeat infection markers showed an increase. A follow-up head CT on the 9th day after admission showed expansion of the hypodense lesion involving the left periventricular region, basal ganglia, thalamus, cerebral peduncle, and temporal lobe (Figure 1F). On the 10th day after admission, the patient underwent open surgery under general anesthesia with tracheal intubation: resection of the left supratentorial deep lesion, internal decompression, and insertion of an intracranial pressure monitoring probe (ICP). Intraoperative frozen section analysis revealed no tumor cells. Brain tissue was sent for metagenomic next-generation sequencing (mNGS). Postoperatively, the patient was drowsy, and both pupils had sluggish responses to light stimulation. Considering the possibility of an intracranial infectious lesion, meropenem and vancomycin were administered. Given the open surgery involving the cortex-basal ganglia region and the infectious nature of the lesion, this patient was considered at risk for developing postoperative epilepsy. Accordingly, sodium valproate was immediately started for seizure prophylaxis. Because of elevated ICP (15 cmH2O), the mannitol dosage was increased to 125 ml every 6 h. The final pathology report confirmed the absence of tumor.

On the 11th day after admission, mNGS identified *Nocardia farcinica* infection. Antimicrobial therapy was then switched to imipenem, amikacin, and sulfamethoxazole. On the 12th day after admission, ICP monitoring indicated that intracranial pressure was essentially normal (13 cmH20), but the CT scan still revealed midline shift. Medications for controlling intracranial pressure were continued and gradually reduced in dosage.

Thirteen months after symptom onset, the patient developed itchy and congested eyes. An ophthalmology consultation was obtained, and conjunctivitis was diagnosed. The consulting ophthalmologist empirically prescribed levofloxacin eye drops, which did not result in clinical improvement. Subsequently, the clinical pharmacology service was consulted. Given the high likelihood of *Nocardia farcinica* infection–despite the patient’s refusal to undergo culture or targeted next-generation sequencing (tNGS) testing–the consulting pharmacologist recommended switching to amikacin eye drops, which led to rapid symptomatic relief.

The patient completed a full course of anti-infective therapy and continued rehabilitation. The follow-up head CT showed that the areas of low-density lesions in the left basal ganglia, thalamus, frontal lobe and insular lobe had all decreased (Figures 1G–J). The follow-up chest CT indicated that the pulmonary infection was gradually alleviating (Figures 2B,C). At follow-up, lower limb function had improved, allowing ambulation with crutches. Although right upper limb deficits persisted, the patient could perform activities of daily living such as face washing and tooth brushing independently.

## Discussion

*Nocardia farcinica* is a rare opportunistic pathogen, primarily affecting immunocompromised individuals. Brain abscesses caused by this organism are uncommon but associated with high mortality. Prompt administration of sensitive antibiotics, such as sulfamethoxazole and amikacin, is essential for treating central nervous system infections due to *Nocardia farcinica*. However, early diagnosis remains challenging because of nonspecific clinical symptoms (9, 10). Metagenomic next-generation sequencing (mNGS) offers a precise and efficient method for pathogen identification, thereby facilitating early and accurate diagnosis (11, 12). Early detection of the pathogen via mNGS from sputum, cerebrospinal fluid, or brain tissue can guide timely antibiotic therapy, potentially reducing disability, mortality, and healthcare costs (13).

A comprehensive medical history (encompassing occupational exposure, medication use, past trauma, and surgical history) is highly valuable in diagnosing brain abscesses. The patient had been exposed to a high bacterial load in her work environment and had been on oral hormone therapy, which likely compromised her immune system, thereby increasing her susceptibility to *Nocardia* infection. Notably, imaging revealed concurrent infections in both the lungs and the brain. We hypothesize that *Nocardia* entered through the respiratory tract, causing a pulmonary infection, which then disseminated intracranially to form brain abscesses, and subsequently spread to the conjunctiva, resulting in conjunctivitis.

Although a small number of Gram-negative bacilli and Gram-positive cocci (coccoid and chain-like) were detected in the sputum culture and smear after admission, the reason why *Nocardia* (filamentous Gram-positive bacillus) were not detected at that time may be that culturing *Nocardia* requires a long time (averaging approximately 18 days), and the positive rate of sputum culture is low (30%), leading to a false negative result and delayed diagnosis. It was not until intracranial mNGS indicated *Nocardia* infection that sensitive antibiotics were initiated. The patient’s white blood cell count, serum procalcitonin, and other inflammatory markers rapidly returned to normal. However, she refused to undergo sputum targeted next-generation sequencing (tNGS) examination or repeat sputum culture and smear. Therefore, we could only speculate based on factors such as the patient’s immunosuppressive condition, working environment, lung imaging features, the significant efficacy of sensitive antibiotics, and the low positive rate of *Nocardia farcinica* in sputum.

Although a few cases of *Nocardia* conjunctivitis have been reported (14, 15), no prior cases of *Nocardia* brain abscess coexisting with conjunctivitis have been documented. In this case, although the patient was receiving oral amikacin, conjunctivitis still developed. Once topical amikacin eye drop was added, however, the symptoms resolved rapidly. To identify the reasons, we conducted a multidisciplinary discussion. We speculate that oral administration may have resulted in insufficient drug concentration in the bulbar conjunctiva. This suggests that topical amikacin may be a more effective treatment for *Nocardia*-related conjunctivitis.

This is the first reported case of a *Nocardia farcinica* brain abscess localized in the basal ganglia, with concurrent lung and conjunctival involvement. Previous reports of *Nocardia*-related brain abscesses typically involved regions such as the frontoparietal (16), temporo-occipital (17), parieto-occipital lobes (18) or the cerebellum (19) and presented with symptoms including headache, dizziness, aphasia, or seizures. Brain abscesses in the basal ganglia have been documented with other pathogens, such as *Providencia rettgeri* (20) and *Staphylococcus aureus* (8). Although hemiplegia due to a *Nocardia* brain abscess with pulmonary infection has been reported, the lesion in that case was situated in the precentral gyrus (21). Basal ganglia abscesses caused by *Nocardia* are rare and can be difficult to distinguish from ischemic stroke or brain tumor (6–8). Both cerebral infarcts and abscesses may show low density on MRI, restricted diffusion on DWI, and reduced ADC values. Patients presenting with basal ganglia abscesses and hemiplegia may be misdiagnosed with acute cerebral infarction and inappropriately treated with thrombolysis. Therefore, in addition to imaging, other diagnostic methods such as mNGS should be employed as early as possible.

In summary, the diagnosis, treatment, and rehabilitation of this case presented both challenges and favorable outcomes. The patient presented outside the thrombolytic window for ischemic stroke, which avoided inappropriate thrombolytic therapy. As hemiplegia progressed and the basal ganglia lesion expanded, surgical intervention was performed in a timely manner. Additionally, *Nocardia* was identified via mNGS of brain tissue, enabling prompt initiation of targeted antibiotics. Finally, a comprehensive rehabilitation program was fully implemented to improve limb mobility and activities of daily living, achieving a satisfactory functional recovery. Nevertheless, this case also has limitations. Although we adjusted the treatment plan dynamically based on imaging progression within a short period of time, we failed to promptly identify the lesion with a “non-typical arterial distribution” on the initial head MRI plain scan. In the treatment of acute ischemic stroke, while “time is brain” emphasizes rapid decision-making, it can also lead clinicians to overlook atypical imaging findings. Moreover, in the differential diagnosis, the patient’s immunosuppressive state was not given due attention. Additionally, the absence of culture and sensitivity data from the brain abscess represents a limitation. The lessons learned from this case are as follows: First, clinicians need to have a deeper understanding of neuroimaging, especially vascular distribution and lesion patterns. Second, a detailed collection of medical history and medication history is crucial for differential diagnosis. Third, in immunocompromised patients presenting with focal neurological dysfunction, a higher index of suspicion for intracranial infections (such as *Nocardiosis*) is warranted, rather than focusing solely on cerebrovascular events. Finally, for future cases of brain abscess, we recommend performing mNGS in parallel with routine culture and susceptibility testing.

## Data Availability

The original contributions presented in this study are included in this article/supplementary material, further inquiries can be directed to the corresponding author.
